# Evaluation of the breathing mode by infrared thermography^☆^^☆☆^

**DOI:** 10.1016/j.bjorl.2023.101333

**Published:** 2023-09-25

**Authors:** Yasmim Carvalho Telson, Renata Maria Moreira Moraes Furlan, Matheus Pereira Porto, Rafael Augusto Magalhães Ferreira, Andréa Rodrigues Motta

**Affiliations:** a Universidade Federal de Minas Gerais (UFMG), Departamento de Fonoaudiologia, Belo Horizonte, MG, Brazil; b Universidade Federal de Minas Gerais (UFMG), Departamento de Engenharia Mecânica, Belo Horizonte, MG, Brazil

**Keywords:** Thermography, Respiration, Mouth breathing, Nose, Mouth

## Abstract

**Objectives:**

To analyze breathing modes with infrared thermography.

**Methods:**

Cross-sectional observational exploratory study conducted in 20 female participants with a mean age of 26.0-years. The thermograms were made following the principles of the American Academy of Thermology and the Brazilian Thermology Society. The camera FLIR A315 (FLIR Inc., Santa Barbara, CA) was used for the tests. The recordings consisted of the participants breathing normally through the nose for 2 min and simulating oral/oronasal breathing for another 2 min. The thermograms were analyzed with the FLIR Tools software. An ellipse was placed between the nostrils and the lip commissures to obtain the mean temperatures. The collection was made by two independent researchers, and the normalized non-dimensional temperature was calculated.

**Results:**

The temperature in nasal breathing is higher than in oral/oronasal breathing both for inhaling and exhaling when measured in the region of the mouth. The exhaling temperatures were higher than the inhaling ones in oral/oronasal breathing (through the nose and the mouth) and nasal breathing (only through the nose). The temperature difference between exhaling and inhaling (ΔT) was greater in oral/oronasal breathing when measured in the region of the mouth.

**Conclusion:**

The thermographic assessment of breathing modes may be made by comparing the mean temperatures of the mouth, using an ellipse.

**Level of evidence:**

Study without consistently applied reference standards.

## Highlights

• Physiological changes in temperature during the respiratory cycle.
• Inspiration, there is cooling of the nose.
• Expiration, heating.
• Thermography as a non-invasive respiratory mode assessment tool.

## Introduction

The breathing mode encompasses the path traveled by the airflow in the upper airways during breathing.^1^ It is a relevant factor influencing on the balance of the orofacial myofunctional system.^2^ Mouth breathing is the total or partial change of nasal airflow to the mouth, usually associated with obstructions in the nose or nasopharynx, in addition to allergic issues.^3^ However, even after the clearing of the obstruction, it may remain as a habit, and for that reason, the speech-language therapy action in the altered function has key importance for the prognosis and good clinical evolution of the patient.^3^

Within the area there is still no objective “gold standard” technique to evaluate the breathing mode, many times done in a subjective way and depending on the report of the signs and symptoms by the patient/caregiver.^4^ A reliable, cost-effective, and non-invasive objective measure of nasal airflow still remains unknown.^5^

Infrared thermography is known to be a non-contact and non-invasive method.^6^ It has been used in different areas of knowledge, among them the health area, for being an equipment capable of detecting the temperature variations involved in the changes of some physiological processes.^6^

**Figure 1:**
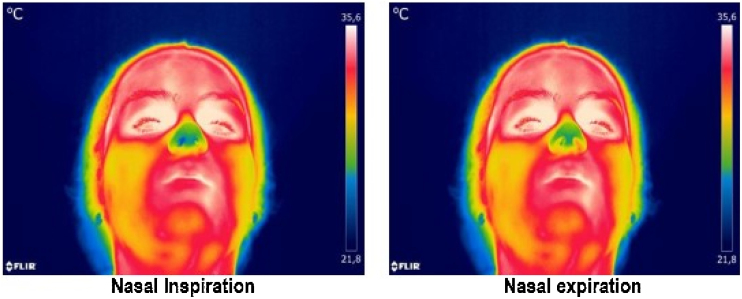
Thermograms selected with the experimental apparatus, during inspiration (right) and expiration through nose (left). In the scale used, the highest temperatures (close to 35.6°) are represented in red, whereas temperatures close to 21.8° are represented in blue.

Studies have employed thermography in the evaluation of breathing, mainly using it as a technique to quantify the respiratory flow or rate.^7^, ^8^, ^9^, ^10^, ^11^, ^12^, ^13^, ^14^, ^15^, ^16^, ^17^ A recent study also aimed to determine nasal functionality by means of this method and obtained promising results.^5^ All these research pieces are based on the physiological changes represented by the respiratory cycle, since during inspiration there is the inhalation of the environmental air with a drop in the temperature of the nose and/or mouth, and during expiration the opposite process is observed: there is the release of air from the lungs and, therefore, the heating of the nose and/or mouth^7^, ^8^ (Fig. 1).

Based on the aforementioned assumptions, this research has as its main objective to analyze the breathing mode by means of infrared thermography. In that way, it will be possible to develop a protocol of collection and analysis of the cutaneous temperatures of the nose and mouth during nasal breathing and during oral/oronasal breathing, helping the speech-language clinical practice through an objective and efficient evaluation of the breathing mode.

## Methods

This research is a cross-sectional observational study of exploratory type. It was developed with self-declared nasal breathing participants at the Functional Health Observatory in Speech-language Therapy at the UFMG (Federal University of Minas Gerais) School of Medicine. All the research participants were speech-language therapists or speech-language therapy students in the clinical cycle, and, therefore, had knowledge about the physiology of breathing and the clinical signs involving mouth breathing. Data collection was only started after approval by the Institution’s Research Ethics Committee by the following opinion: CAAE 32465518.0000.5149 and all signed the informed consent form.

Among the inclusion criteria for the study were: being 18-years of age or older; being a nasal breather; not presenting any physical, neurological, and/or cognitive alteration that would hinder or make it difficult to collaborate during the exam.

Regarding the exclusion criteria for the participants, the following were established: the use of bandages on the skin and/or other factors that would prevent exposure to a temperature-balanced environment; physical exercise, acupuncture, massages, or having undergone electrical stimulation, saunas, or prolonged exposure to the sun up to 24 h before the measurements were taken; the use of bronchodilators and vasoactive medications; the presence of fever, allergic rhinitis, flu and/or cold symptoms on the day of the tests; the presence of orofacial pain; men with facial beards, and women in the fertile or menstrual period.

The following three steps were observed for the selection of participants and classification of the breathing mode, in addition to self-reporting of nasal breathing: evaluation of the signs and symptoms of mouth breathing, lip seal evaluation, and evaluation of orofacial muscle tone.

To evaluate the signs and symptoms a questionnaire containing major and minor signs for mouth breathing was applied among the participants.^18^ Were considered suspected cases of oral/oronasal breathing and, therefore, excluded from the research the participants that presented at least two major signs or a major sign associated with two minor signs.^18^ Afterwards, the lip-sealing ability was evaluated for two minutes. To be considered a possible nasal breather the participant should be able to keep the lip seal without perioral tension for the proposed time of two minutes. Finally, the tone of the right and left masseter muscles, the orbicularis oris, as well as the right and left buccinator muscles were checked by subjective palpation. Only the participants who passed the three proposed stages were selected for the study, that is, had less than two major signs for mouth breathing or a major sign associated with two minor signs, had a lip seal for two minutes or more, besides adequate tonus for the masseter muscles bilaterally, orbicular of the lips and right and left buccinator muscles.

After the selection of participants by means of the described selection criteria, the final sample was composed of twenty nasal breathing women, totaling 240 thermograms.

The thermography records were performed following all topics recommended by the American Academy of Thermology and the Brazilian Association of Thermology.^19^ The room temperature was controlled (20° ± 1 °C) and, together with relative humidity (which did not exceed 70%), was measured using HTH-426A — HOMIS thermo-hygrometer. The participants remained in the room from 15 to 20 min before the measurements for temperature stabilization, without direct exposure to the ventilation system. A recommendation list was sent to the participants one day before the measurements. During the collection they were asked to remove accessories such as earrings, necklaces, and piercings, as well as to tie up their hair. The assumed emissivity was 0.98 which corresponds to the value of human skin.^20^

A FLIR A315 camera (FLIR Inc., Santa Barbara, CA) and a FLIR 18 mm lens were used for the tests. The equipment was positioned on a tripod at a distance of approximately one meter from the face. The volunteer was seated on a fixed chair with a back resting against the wall. The thermal camera was frontally positioned on a tripod with an angle between 80° and 90° for better viewing of the nose and mouth simultaneously. A grid panel was also inserted behind the participant to allow head alignment during image recording. A sequence of 5 frames per second was used to take the videos.

The tests consisted of three different moments, initially the nasal breathing was requested for two minutes of recording. Subsequently, the patient was asked to simulate oral/oronasal breathing as a form of training, therefore without filming. Only in the third stage, after training, the participant was recorded for two minutes during oral/oronasal breathing.

**Figure 2:**
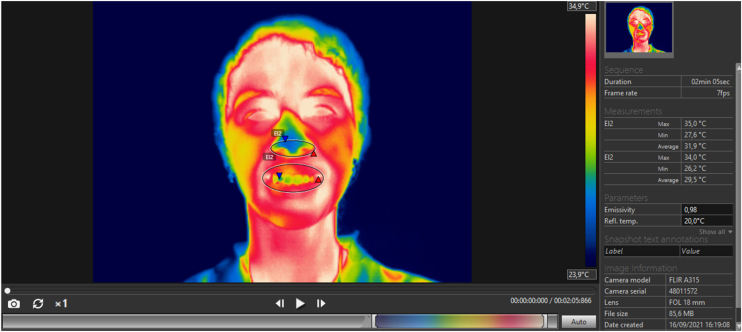
Manual selection of ROIs in the Flir Tools software using an ellipse.

After data collection, the nose and mouth temperatures were obtained and analyzed. To obtain this information the Flir Tools software was used. An ellipse was positioned between the lip commissures, the cupid’s bow and the lower limit of the lower lip and chin to obtain the thermal values of the mouth. To obtain nasal temperature, another ellipse was positioned around the nostrils, the nose apex, and the nasolabial angle (Fig. 2). The choice of the ellipse as a tool for selecting the regions of interest was due to the robust results obtained in a pilot study previously carried out, and because it adapts more perfectly to the anatomy of the nose and mouth. The average temperatures of the selected regions were then collected for both inspiration and expiration. The use of the average temperature in this research is also due to the satisfactory results obtained in a previous analysis.

For the selection of the video images the following procedures were performed: all the two-minutes videos were divided into three moments of 40 s and in each of these moments, two distinct images were selected, that is, one image for inspiration and one image for expiration. Therefore, six images (three of inspiration and three of expiration) were selected from the nasal breathing videos and six images from the oral/oronasal breathing videos (three of inspiration and three of expiration), totaling twelve images per participant. Therefore, the final data analysis was composed of 240 thermal images.

The criterion used to select the best inspiration image was based on the physiology of breathing, and the selected image was the one that presented the lowest minimum temperature. Similarly, we chose the one with the highest maximum temperature for the selection of the expiration image. The collection of temperatures was performed by two independent evaluators, as well as replicated by the same evaluator for agreement analysis.

As the infrared thermography technique measures the absolute temperature, it is influenced by the individual and local temperatures. To correct for these factors, the normalized non-dimensional temperature was calculated, which consists of quantitative numerical values ranging from 0 to 1.^21^ To this end, the tympanic temperatures of all participants were measured with a Prosnubl thermometer, model NO EFT 161, and the ambient temperatures of the room at the time of the exam were obtained for the following calculation: $\theta =\frac{T-T_{\infty }}{T_{b}-T_{\infty }}$.

Where θ is the corrected non-dimensional temperature; *T* the temperature of the assessed surface (nose and mouth); *T*_∞_ the ambient room temperature at the time of the examination; and *T*_b_ the participant’s tympanic temperature.

Data were analyzed using the data analysis program IBM SPSS version 25. To test the distribution of the continuous variables, the Shapiro–Wilk test was applied, indicating a non-symmetric distribution (<0.05). Comparisons between the corrected non-dimensional nose and mouth temperatures in nasal breathing and oral/oronasal breathing during inspiration and expiration and the ΔT difference (expiration‒inspiration) of nasal and oral/oronasal breathing were done using Wilcoxon’s test. Three temperature measurements were collected for each variable studied, so for statistical analysis the median of these three measurements was adopted as the parameter analyzed throughout the study. The significance level adopted was 5%.

The intra evaluator and inter evaluator agreement analysis was done by means of the Intraclass Correlation Coefficient (ICC), since these are continuous measurements. In this analysis we used only the temperature data obtained from the mouth due to the statistically significant values obtained in this study. All analyses showed desirable agreement, i.e., above 0.800 (Table 1).

**Table 1:** Intra and inter evaluator agreement of mouth temperatures.

		ICC — intra^a^	ICC 95%	*p-*Value	ICC — inter^a^	ICC 95%	*p*-Value
Inspiration	NB	0.994	(0.984‒0.988)	0.000	0.991	(0.978‒0.997)	0.000
	OB	0.887	(0.714‒0.955)	0.000	0.922	(0.803‒0.969)	0.000
Expiration	NB	0.988	(0.970‒0.995)	0.000	0.990	(0.975‒0.996)	0.000
	OB	0.899	(0.744‒0.960)	0.000	0.897	(0.739‒0.959)	0.000

NB, nasal breathing; OB, oral/oronasal breathing; ICC, Intraclass Correlation Coefficient.

a Wilcoxon test.

## Results

When comparing nose temperatures between Nasal Breathing (NB) and Oral/Oronasal Breathing (OB) both at inspiration and expiration, no significant differences were observed (Table 2). Statistically significant differences were obtained in the comparison of mouth temperatures between nasal breathing and oral/oronasal breathing both at inspiration and expiration (Table 2), resulting in higher temperature of nasal breathing.

**Table 2:** Comparison of corrected dimensional temperatures of the nose and mouth in nasal breathing and oral/oronasal breathing.

Region of interest	Inspiration			Expiration		
	NB	OB	*p-*Value^a^	NB	OB	*p*-Value^a^
Nose	0.58 (0.45‒0.64)	0.56 (0.45‒0.66)	0.520	0.60 (0.48‒0.68)	0.57 (0.48‒0.68)	0.654
Mouth	0.79 (0.76‒0.84)	0.66 (0.63‒0.69)	**0.002**	0.79 (0.76‒0.83)	0.68 (0.65‒0.72)	**0.002**

NB, nasal breathing; OB, oral/oronasal breathing.

Values in bold represent the p value less than or equal to 0.05.

a Wilcoxon test.

Table 3 shows the comparison between nose and mouth temperatures in inspiration and expiration. The values obtained in the nose both during nasal breathing and oral/oronasal breathing showed a difference, being the expiration values higher. As for the measurements taken in the mouth, there was a difference with statistical significance only during oral/oronasal breathing, and the temperatures in expiration were higher than in inspiration.

**Table 3:** Comparison of the corrected non-dimensional temperatures during inspiration and expiration.

Breathing mode	Region of interest	Inspiration	Expiration	*p*-Value^a^
NB	Nose	0.58 (0.45‒0.64)	0.60 (0.48‒0.68)	**0.000**
	Mouth	0.79 (0.76‒0.84)	0.79 (0.76‒0.83)	0.790
OB	Nose	0.56 (0.45‒0.66)	0.57 (0.48‒0.68)	**0.022**
	Mouth	0.66 (0.63‒0.69)	0.68 (0.65‒0.72)	**0.000**

NB, nasal breathing; OB, oral/oronasal breathing.

Values in bold represent the p value less than or equal to 0.05.

a Wilcoxon test.

Comparing the temperature difference between expiration and inspiration (ΔT) in nasal and oral/oronasal breathing allows to notice differences with statistical relevance in the comparison with the temperatures in the mouth region (Table 4).

**Table 4:** Comparison of ΔT (expiration‒inspiration) of nasal and oral/oronasal breathing.

Region of interest	ΔT NB	ΔT OB	*p*-Value^a^
Nose	0,03 (0,05; 0,01)	0,01 (0,03; 0)	0,053
Mouth	0 (0,01; 0)	0,02 (0,05; 0,02)	**0,000**

ΔT, difference between expiration and inspiration; NB, nasal breathing; OB, oral/oronasal breathing.

Values in bold represent the p value less than or equal to 0.05.

a Wilcoxon’s test.

## Discussion

The results obtained illustrate the applicability of infrared thermography for the evaluation and classification of the breathing mode. It is important to emphasize that the analysis used the non-dimensional normalized temperature, which allows the interpretation of thermal values without the metabolic influence of each individual, as well as the environmental temperature of the room at the time of testing, thus making the analyzed results more reliable.^22^

According to the findings we can consider that the mouth can be used as a Region of Interest (ROI) for thermographic analysis of the breathing mode.

When comparing the temperatures during nasal breathing and oral/oronasal breathing, we noticed a statistically significant difference when comparing the temperatures of the mouth, both during inspiration and expiration, being higher during nasal breathing. This result may be associated with the fact that in nasal breathing there is no contact with atmospheric air, making it possible to keep the body temperature stable in the mouth. In oral/oronasal breathing, on the other hand, air intake and outlet seem to be able to decrease the body temperature.

Results with statistical relevance for the mouth were also observed in the analysis of the temperature difference between expiration and inspiration (ΔT). In this case the ΔT of oral/oronasal breathing was higher than that of nasal breathing, revealing greater airflow through the mouth during oral/oronasal breathing.

The comparison between inspiration and expiration temperatures showed statistically significant differences in the temperatures collected in the mouth and nose during oral/oronasal breathing, and in the temperatures collected in the nose during nasal breathing. In all those three situations the values in expiration were higher than in inspiration. This fact is concurrent with the literature, since during expiration the warm air from the lungs is exhaled, thus warming the nose and mouth, while during inspiration there is cooling of the nasal vestibule and mouth.^8^, ^9^ As expected, there were no differences in the temperatures of the mouth during nasal breathing, since there was no airflow through the mouth, and all participants had their lips sealed, so thermography was effective in detecting temperature changes during breathing.

This research has great importance for Orofacial Motricity practice, since it presents an objective, simple, non-invasive and visual technique for defining the breathing mode, facilitating its evaluation, which is often subjective and lacking a “gold standard” procedure. Moreover, it is known that mouth breathing is a frequent symptom in childhood,^22^ therefore the presence of a precise equipment for rapid evaluation of the breathing mode is of great relevance for the speech-language therapy clinical performance.

However, despite the satisfactory results, this research has some limitations. The sample size of the study is relatively small, i.e., it was composed of twenty participants, corresponding to a total of 240 thermograms; therefore, further investigations with a larger sample size are suggested. Moreover, to evaluate oral/oronasal breathing, data were collected from nasal breathing subjects simulating this breathing mode; therefore, data from patients with a multidisciplinary diagnosis of oral/oronasal breathing were not used.

This research is innovative and no other studies with similar objective were found, only one recent study^5^ that evaluated functional noses of substantially obstructed noses comparing the temperatures of the nostrils with subjective scores of the NOSE scale (Nasal Obstruction Symptom Evaluation) that evaluates symptoms of nasal obstruction.^23^ For the purposes of future research, we suggest composing the sample with mouth-breathing subjects and investigating a cutoff point in temperature differences that may allow to determine the breathing mode by means of infrared thermography.

## Conclusion

It was possible to perform the thermographic evaluation of the breathing mode by comparing the mean temperatures of the mouth, using an ellipse to define the region of interest to be analyzed. The calculation of the corrected non-dimensional temperature was relevant in the analyses since it allowed correcting the influence of the environmental temperature and the core temperature of the individual during thermographic evaluations.

## Authors’ contributions

YCT was responsible for preparing the research design, collecting, and analyzing data, and writing the manuscript. RMMM, MPP, RAMF and ARM performed the overall guidance of the work, supervising the preparation of the research project, data analysis and writing of the article.

## Funding

None.

## Conflicts of interest

The authors declare no conflicts of interest.
